# BioPsychoSocial Medicine: a new open access journal

**DOI:** 10.1186/1751-0759-1-1

**Published:** 2007-01-10

**Authors:** Yoshihide Nakai

**Affiliations:** 1 President, Japanese Society of Psychosomatic Medicine, Japan; 2 Professor, Department of Psychosomatic Internal Medicine, Kansai Medical University, Japan

## 

We are pleased to announce the launch of *BioPsychoSocial Medicine *[1], an open access, online, peer reviewed journal that considers articles on all aspects of the inter-relationships between the biological, psychological, social, and behavioral factors of health and illness.

BioPsychoSocial Medicine is the official journal of The Japanese Society of Psychosomatic Medicine [2], which was established on November 30, 1959, and now has over three thousand seven hundred members. The Japanese Society of Psychosomatic Medicine is an interdisciplinary academic society consisting mainly of physicians, gynecologists, psychiatrists, pediatricians, dermatologists, dentists, researchers of basic medical science, and psychologists. The society publishes a monthly Japanese language academic journal, the *Japanese Journal of Psychosomatic Medicine *[3], the latest issue being Vol. 46 No. 12.

With the support of psychiatrists, Japanese psychosomatic medicine specialists have aspired to promote the development of clinical medicine, education, and research into biopsychosocial medical science targeting the physical disorders of each branch. Our members' wishes to transmit their results to the world have been realized through the publication of an English language journal, *BioPsychoSocial Medicine*.

We welcome the contributions of psychosomatic medicine scientists from around the world, as well as from those in Japan, and have high expectations that by publishing high-quality research papers on biopsychosocial medical science and by providing opportunities for communication among the psychosomatic medicine scientists of the world, *BioPsychoSocial Medicine *will become an important journal in its field. We look forward to receiving your submissions.

## Competing interests

Yoshihide Nakai is an Advisory Board member of *BioPsychoSocial Medicine *and President of the Japanese Society of Psychosomatic Medicine.
